# Food Supplements and Their Use in Elderly Subjects—Challenges and Risks in Selected Health Issues: A Narrative Review

**DOI:** 10.3390/foods13162618

**Published:** 2024-08-21

**Authors:** Maria João Campos, Magdalena Czlapka-Matyasik, Angelina Pena

**Affiliations:** 1 LAQV-REQUIMTE, Laboratory of Bromatology, Pharmacognosy and Analytical Sciences, Faculty of Pharmacy, University of Coimbra, 3000-295 Coimbra, Portugal; 2 Department of Human Nutrition and Dietetics, Poznan University of Life Sciences, 60-624 Poznan, Poland; magdalena.matyasik@up.poznan.pl

**Keywords:** food supplements, malnutrition, elderly, polypharmacy, pharmacists, interactions, risk

## Abstract

The European population is ageing. Food Supplements (FSs) are foods with particular characteristics, consumed by elderly people for various purposes, including combating nutritional deficits. Their consumption in this age group, associated with a high prevalence of polypharmacy, can enhance interactions. Potential drug-food (or food supplements), drug-drug interactions and polypharmacy are common health issues among older adults. The prevalence of polypharmacy is high, and preliminary data also indicate that there is significant FS use, increasing the risk of the duplication of therapies and various adverse reactions as well as drug–FS and FS-FS interactions. Therefore, the intervention of health professionals in mitigating these risks is essential. This review highlights and discusses the association between FSs, polypharmacy, and adverse reactions due to the risk of potential interactions between these products. Moreover, it also provides current scientific evidence regarding the use of FSs by the elderly. A review of the challenges, advantages, and risks of using FSs in elderly people who are malnourished and/or polymedicated, focusing on the good practises needed to support healthy ageing, is presented. In this regard, this paper aims to help health professionals better deal with the issue of the use of multiple FSs and polypharmacy, overcome the malnutrition problem, and improve the health and well-being of older adults.

## 1. Introduction

In 2019, the United Nations stated that population ageing is about to become one of the most significant social transformations of the current century. Forecasts indicate that the population of elderly people aged 60 or over will double by 2050 and more than triple by 2100, rising from 962 million in 2017 to 2.1 billion in 2050 and 3.1 billion in 2100 [1].

By 2024, it is estimated that the population of individuals aged over 65 years will outnumber those under the age of 15 in the World Health Organization (WHO) European Region [2]. Worldwide, healthy life expectancy at birth has improved by 5.36 years from 58.3 years in 2000 to 63.7 years in 2019 [3].

As Europe’s population ages, increased life expectancy leads to a rise in age-related diseases. Older adult well-being demands multidisciplinary strategies to support healthy ageing. First, the elderly consume fewer nutrients, and even those who correctly ingest them may not absorb nutrients due to physiologic changes, leading to a deficiency of essential vitamins and minerals [4]. Second, age-related diseases that result from gradual cellular damage must be addressed. Finally, the prescription and consumption of medicines and other health products have increased amongst the geriatric population, who often take multiple medications. Consequently, they are more likely to experience adverse pharmacological effects [5,6,7]. On average, it is estimated that one in ten patients is susceptible to an adverse event while receiving hospital care in high-income countries [8].

Moreover, the results of the latest investigation on this topic suggest an association between malnutrition and polymedication, which has been recognised as a global public health problem in older adults [1] and could be avoidable.

Therefore, ensuring the best diet possible for older people and using FSs to complement their diet is crucial, effectively bridging the gap between malnutrition status and achieving the recommended nutritional intake.

At the same time, some data lead us to believe that a significant percentage of individuals over 60 consume FSs, but little is known about the characteristics of FSs’ elderly users [9].

To achieve well-being, older adults are also easily influenced by friends or television commercials potentiating the consumption of FSs. Food (including FSs)–drug interactions risk should be highlighted. With an increase in age, the prevalence of multiple diseases also increases, leading to polypharmacy. The poor communication between patients and physicians and the lack of health literacy may contribute to unreported FS use. Therefore, these interactions are difficult to detect.

European legislation about FSs is only partially harmonised. The legislation regulates vitamins and minerals and their sources, which can be used in the manufacturing of FSs [10]. For other ingredients, the European Commission (EC) has established harmonised rules to protect consumers against potential health risks. FSs cannot replace a regular and healthy diet but aim to increase and promote human health [11,12,13]. When advising their patients on these products, the tools to support health professionals are practically non-existent, and most information about FSs is obtained from the companies’ web pages.

The transposition of European Directive 2002/46/EC into national laws does not guarantee that pharmacists and other health professionals will provide duly informed advice on these products, as happens in Portugal. However, some countries, such as France, Italy, and Belgium, have implemented mild procedures to ensure safer advice on these products [11,12,13].

Currently, there is growing concern about the misuse of FSs, their potential for interaction with medicines, and their confusion with them. This misunderstanding starts with the definition of the name of the products. Medicine is available in Portugal, France, Spain, Korea, the United States of America, and worldwide. However, a multivitamin can be referred to as an FS in Portugal and a dietary supplement in the United States of America. These differences in designations make the research challenging. For this investigation, dietary supplements and FSs were considered the same, and they are referred to as FSs, as their definitions overlap [11,12,14]. Additionally, FSs can be sold in pharmacies, dietetic stores, supermarkets, phone sales, or catalogues, or on the Internet. This range of points-of-sale raises the problem of a lack of pharmacist advice.

This issue alone is confusing. When evaluating the FSs associated with the elderly population, it becomes even more complex. Consequently, there should be an awareness of the importance of this area of study, which should be considered a very relevant aspect of healthy ageing.

Last October, the Lisbon Outcome Statement “Unlocking the future of healthy ageing” emphasised the need for practises to support healthy ageing and highlighted the importance of a collective effort from all sectors to promote healthy ageing. One of the key priorities focuses on preventing health issues and providing data and evidence to support policy decisions informed by evidence-based practises [15].

This paper aims to comprehensively review the potential association between polypharmacy and FSs, especially in the elderly and malnutrition, and emphasise the role of health professionals in facilitating knowledge exchange at regional and global levels to better deal with (1) FS consumption in the elderly, (2) emphasising the benefits and risks of FS use in this age group, (3) polypharmacy in the elderly, (4) overcoming the malnutrition problem, and (5) improving the health and well-being of older people.

## 2. Methodology

**Figure 1:**
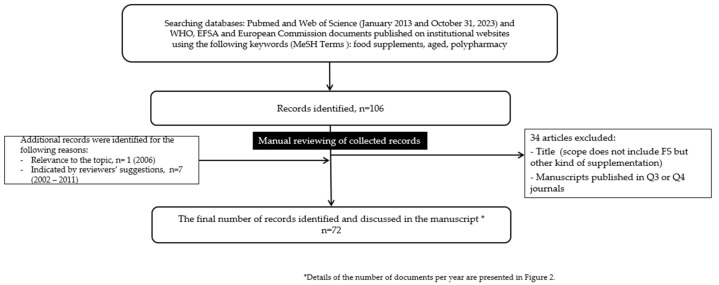
Methodology.

**Figure 2:**
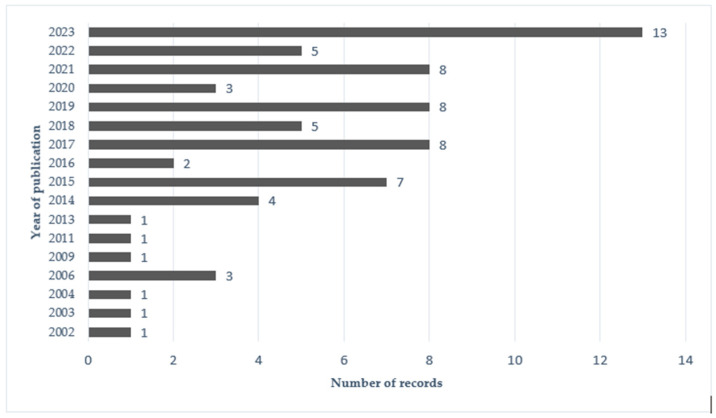
Number of Records per year of publication.

This review provides an overview of recent publications on FS consumption in the elderly and its relationship with some health issues, namely malnutrition and polypharmacy. Figure 1 represents the methodology steps and Figure 2 an overview of the number of records per year.

## 3. Results

### 3.1. Elderly

There is some controversy in defining the age at which a person should be considered elderly. In pharmacotherapeutic terms, some even advocate for not defining a chronological age but adapting it to the person’s metabolic age [16]. The WHO considers any individual over 60 an elderly or older person. This definition applies to most industrialised countries and the United Nations, and it was the age considered in this research [17]. Nevertheless, in some studies published in the scientific literature, an age of 65 or more is considered, potentially producing biassed results.

Increased life expectancy has led to an increase in chronic diseases, such as cardiovascular and neurodegenerative diseases and diabetes, which include an oxidative stress component in the underlying diseases [5]. Multimorbidity is associated with a low quality of life and disabilities and usually requires treatment with multiple medicines, which increases the risk of adverse medical events and leads to an even greater risk of death [1].

Age-related limitations occur in physical, mental, and/or social functions as a consequence of acute and/or chronic illnesses in combination with neurodegenerative changes typical of this stage of life. The ability to perform the basic activities of daily life independently is jeopardised or even lost. To avoid partial and total loss of independence, the elderly need a holistic approach that includes rehabilitation and physical, psychological, and social care [18].

The elderly population undergo physiological and pharmacologic (pharmacokinetic and pharmacodynamic) changes that make them particularly susceptible to presenting secondary or adverse medication effects, even with commonly used medicines [6,7]. Therefore, polypharmacy can put the patient at risk of medicine-related adverse events and interactions when not used correctly [1].

It should also be highlighted that adequate nutritional status is essential in preserving health status and preventing or delaying the progression of age-related diseases in older adults.

### 3.2. Polypharmacy

Polypharmacy is defined by the WHO as “the administration of many drugs at the same time or the administration of an excessive number of drugs” or “the concurrent use of multiple medications” [6,19]. In 2021, a review indicated that one hundred and forty-three definitions of polypharmacy and associated terms were found. Most of them are numerical definitions [6]. Although there is no standard definition, polypharmacy is often defined as the daily routine use of five or more medicines. This is the most commonly used for polypharmacy, with 46.4% (n = 51) of studies using this definition [6,20,21]. It includes over-the-counter or prescription medicines and, in some definitions, traditional and complementary medicine prescriptions, including FSs [19,22].

There is an association between adverse health outcomes and polypharmacy [20,23], including mortality, falls, adverse drug events, drug–drug and food–drug interactions, non-adherence, increased length of stay in hospital, and readmission to a hospital shortly after discharge [20].

In most cases, using multiple medications is clinically appropriate, particularly in the elderly. However, they may be used despite patients having no need for them, which can occur due to imitation. Imitating harmful behaviour patterns, especially abnormal ones, occurs much more frequently than appropriate and healthy ones [24].

However, it is essential for health professionals, namely physicians and pharmacists, to be aware of medication duplication or even inappropriate polypharmacy, which can increase poor patient health outcomes [20].

Interactions between various pharmacologic active substances cannot be ignored, whereas adverse drug reactions could be misdiagnosed. This misinterpretation can lead to the prescription of new drugs, which places the patient at risk of developing additional adverse effects. This process is known as the “prescribing cascade”. Indeed, medication-related problems in older people can be considered a geriatric syndrome [25].

Polypharmacy is a topic that has generated much concern, having been considered by the WHO to be a public health problem [19]. The WHO launched its third Global Patient Safety Challenge: Medication Without Harm in March 2017 to reduce severe, avoidable medication-related harm worldwide by 50% by 2022 [26,27].

#### Prevalence of Polypharmacy

As the prescribing rates continue to increase because of the rise in the older adult population and the availability of many more medications globally, studying the prevalence of polypharmacy is essential to support the definition of strategies to fight this global healthcare problem. Numerous studies have assessed and reviewed the prevalence of polypharmacy in elderly people worldwide, and it is crucial to understand the existing data on populations living in European countries.

Some authors (Guillot et al., 2019) concluded, with their research, that the prevalence of polypharmacy is between 10 and 90% [28], and others between 4 and 96% [29]. This wide range of prevalence is due to different reasons, one of which seems to be related to the different definitions of polypharmacy, since there is no harmonised definition within Europe. Nevertheless, age groups and the geographical locations of the data collection should also be considered. A Portuguese study published in 2019 showed that polypharmacy is present in more than 70% of hospital-admitted elderly patients [22].

To better understand the prevalence variation in European countries, the data reported in the Survey of Health, Aging, and Retirement in Europe (SHARE) database [30], in which polypharmacy is defined as the concurrent use of five or more medications in people older than 65 years, were evaluated. The results ranged from 26.3% to 39.9% across 17 European countries plus Israel. Portugal, Israel, and Czechia have, in this study, the highest prevalence of polypharmacy, at 36.9%, 37.5%, and 39.9%, respectively [30]. Across Europe, 32.1% of older adults take five or more medications per day [30].

Relevant studies about polypharmacy prevalence were analysed and are summarised in Table 1, where it is possible to verify the differences between polypharmacy classifications.

From the data reported in these studies, it is not completely clear that polypharmacy does not include products that are not medicines, such as FSs or oral nutritional supplements. This situation is also limiting in data analysis and needs to be improved.

### 3.3. Malnutrition’s Relationship with FSs and Polypharmacy

When studying the elderly, FS consumption, and polypharmacy, malnutrition is frequently mentioned. It is crucial to explain how this factor influences and is influenced by taking various medications and its relationship with using FSs.

ESPEN (The European Society of Clinical Nutrition and Metabolism) defines malnutrition as “a state resulting from lack of intake or uptake of nutrition that leads to altered body composition (decreased fat-free mass) and body cell mass leading to diminished physical and mental function and impaired clinical outcome from disease” [38].

Malnutrition encompasses both overnutrition and undernutrition. In this review, undernutrition is referred to as malnutrition in the elderly, who face a heightened risk. It is estimated that around a quarter of European older adults are malnourished or at risk of malnutrition [39]. A variety of factors contribute to this, including age-related physiological decline, anorexia, oral health problems, biological changes in the digestive system, chronic diseases, polypharmacy, limited access to nutrient-rich foods, and social conditions. Acknowledging and addressing these risk factors can help ensure that older adults receive the essential nutrients they need to maintain optimal health and well-being [40].

High-income countries, such as Portugal, also report food insecurity in over two-thirds of older populations due to low economic resources, which contributes to the high prevalence of malnutrition [41]. Data from scientific studies in Europe have highlighted unfavourable intakes by older adults of total and saturated fat, sugar, salt, and dietary fibre with low intakes and a suboptimal status of crucial micronutrients such as vitamin D, complex B vitamins, and calcium [42]. Malnutrition predisposes older adults to an increased risk of adverse clinical outcomes [40]. Physically, better nutritional status is associated with better cognitive function and functional capacity in the elderly [43]. Furthermore, a positive association between polypharmacy and malnutrition has been found, but this relationship is not fully defined [21,44,45].

The relationship between polypharmacy and malnutrition is based on several mechanisms. The prolonged use of various medicines leads to anorexia, which can generally cause mild or more severe impairment of the digestive system and, consequently, a deficit in nutrient absorption. Furthermore, many medications have the potential to negatively affect nutritional status by changing the sensory perception of taste, intestinal absorption, and metabolism or inducing the elimination of essential micronutrients. More than 250 drugs have been reported to have adverse effects on patients’ nutritional status. Pharmacists, nutritionists, and other professionals should know the potential effects of individual drugs and polypharmacy on, for example, perioperative nutritional status, and seek to reduce their negative impacts [46].

Age-related physiologic, pathologic, and environmental changes also place older adults at increased risk of polypharmacy and malnutrition. These changes include the increased prevalence of chronic medical conditions, as mentioned before, and reduced senses of thirst and taste, which can increase the risk of fluid and electrolyte imbalances and dry mouth. Moreover, accessing a nutritionally adequate diet is more challenging due to disability and food insecurity (food availability is scarce because of many reasons, including environmental changes, which make correct food intake difficult) [21].

Furthermore, older patients are at even greater risk of adverse effects due to decreased renal and hepatic function; lower lean body mass; reduced hearing, vision, cognition, and mobility; and also sarcopenia [20,47,48]. Subjects with sarcopenia had a greater prevalence of malnutrition [48].

Given the significant prevalence of polypharmacy amongst the elderly population and the evidence indicating its potential impact on malnutrition, it is possible to pursue two complementary strategies to combat this issue. First, an assessment of a more rational prescription, and second, improving the patient’s nutritional status. A healthy personalised diet is fundamental but may not be sufficient, and in these situations, physicians, pharmacists, and nutritionists have to resort to FSs or oral nutritional supplements. Oral nutritional supplements are described as follows: ‘food for particular medical purposes’ means food specially processed or formulated and intended for the dietary management of patients [49]. These products are usually presented in 200/300 mL bottles, so any similarity with the FS classification is pure distraction. Additionally, they are products with a relevant caloric value (up to 50 kcal) [50].

### 3.4. Food Supplements

As previously mentioned, one of the aims of this review is to contextualise and frame the opportunities and risks of the consumption of FSs by the elderly. For a better understanding, it is essential first to consider its definition and, second, to be aware of the lack of European legislation for FSs.

FSs are concentrated sources of nutrients or other substances with a nutritional or physiological effect, which are marketed in small-“dose” form (tablets, liquids in measured doses, etc.). A wide range of nutrients and other ingredients might be present in their composition, including micronutrients, amino acids, fibre, plants and herbal extracts, and others. They are intended to correct nutritional deficiencies, maintain an adequate intake of certain nutrients, or support specific physiological functions. They are not medicines, so they cannot exert a pharmacological, immunological, or metabolic action. Therefore, their use is neither intended to treat or prevent human diseases nor to modify physiological functions. The European Union regulates FSs as food [11,12,13].

Moreover, studies on the prevalence of FS consumption by the elderly in Europe are scarce, and this food category is excluded from European guidelines for malnutrition. Therefore, below, we present some pertinent information and studies conducted worldwide. Some older studies are also included due to the lack of more recent robust studies.

#### 3.4.1. Food Supplements—Consumption Data

Data on the consumption of FSs in Europe are scarce and not recent; however, what exists shows the high use of these products. As far as is known, data from a study performed in 2015 disclosed that the use of FSs was reported by 26.6% of the Portuguese population and was higher in females, adults, and elderly individuals [9]. In 2014, in Belgium, 38.3% of the population (3–64 years) consumed an FS during the year. More women (47.0%) than men (29.1%) consumed FSs. No information has been collected for people over 64 years of age [51]. More recently, in 2022, the Consumer Survey on FSs in the European Union conducted by IPSOS on behalf of FS Europe concluded that almost nine in ten (88%) of the respondents had used an FS at some point in their lives. The reasons for using an FS were also determined, and the use of FSs for general health reasons increased with age [52]. In 2018, a PlantLibra Consumer Survey about Plant Food Supplements (Plant FSs) revealed that interest in Plant FSs in Italy is high and the prevalence of “regular” adult consumers is 22.7%, which is similar to the Portuguese data [9,53]. FS use is also common in the United States of America. Survey results suggest that the prevalence of regular FS use among adults in the United States of America is about 50%, but the overall prevalence of FS use may be closer to two-thirds of the adult population. FS use in adults has been consistently reported to increase with age, income, and education, and within each age group, women are also more likely to use FSs than men [51,54,55,56] It should be noted that the majority of data on FS intake by the elderly are gathered through research on the economic benefit of preventing certain diseases through FS use.

Research conducted in the Netherlands, the United Kingdom, and France based on savings studies conclude that FSs used to prevent some diseases in the elderly, such as osteoporosis and common respiratory tract infections, have cost-saving benefits [57,58,59,60].

#### 3.4.2. Food Supplements Benefits

Eating a healthy, varied diet is essential to optimal nutritional status, but the ability of individuals to achieve this varies considerably depending on income, disability, food access, attitudes, cooking skills, and knowledge. Scientific evidence combined with economic impact studies reveals that topping up diets with supplemented nutrients prevents nutritional deficiency and could lead to significant health benefits due to the beneficial impact on chronic disease risk.

Supplementation is an effective way of bridging the gap between current nutritional status and optimal intakes. Indeed, this is already recognised for certain nutrients, as illustrated by the example of vitamin D supplementation, which is now recommended across the European Union due to the approval of health claims for vitamin D [61,62,63]. Looking at what is happening in the United States of America, the Dietary Guidelines for Americans for 2015–2020 and 2020–2025 state that nutrient needs should be met primarily from nutrient-dense foods. Nevertheless, it also states that in some instances, fortified foods and FSs may be helpful [64,65].

Some professionals and researchers have also addressed the issue of the benefits of oral supplementation through FSs. It is widely known that the process by which this type of product is placed on the market does not require efficacy and safety tests [66]. In addition, European legislation requires the use of health claims approved by the European Food Safety Authority (EFSA). A health claim is any allegation that states, suggests, or implies the existence of a relationship between a food category, a food (e.g., micronutrient or bioactive), or one of its constituents and health. In this sense, the benefits and safety of the claimed active substance are confirmed whenever there is a health claim. When there is no health claim, doubts persist [62,67].

The scarcity of scientific studies supporting FSs’ benefits does not give us an understanding to generalise its effectiveness [47,68]. Entities that develop and manufacture FSs must rely on substances that have health claims approved by the EFSA. Furthermore, the industry must invest in clinical trials of its FS formulations, whose submission to the EFSA allows the approval of a health claim after expert analysis. A recent case is the combination of artichoke leaf dry extract standardised in caffeoylquinic acid, monacolin K in red yeast rice, sugar-cane-derived policosanols, procyanidolic oligomers from French maritime pine bark, garlic dry extract standardised in allicin, d-α-tocopheryl hydrogen succinate, riboflavin, and inositol hexanicotinate, which reduces blood LDL-cholesterol concentrations. The authorised health claim may be used only for FSs which provide 600 mg artichoke leaf dry extract with 30–36 mg caffeoylquinic acid, 500 mg red yeast rice with 2 mg monacolin K, 10 mg sugar-cane-derived policosanols, 20 mg French maritime pine bark extract with 18 mg procyanidolic oligomers, 30 mg garlic dry extract with 0.25 mg allicin, 30 mg α-tocopherol equivalents, 5 mg riboflavin, and 9 mg inositol hexanicotinate divided into three daily doses to be consumed with the largest meals [69].

#### 3.4.3. Food Supplements: Potential Interactions

Health professionals have the information and the knowledge that FSs contain vitamins and minerals and herbal plants or extracts that have a high potential to interact with medicines. Nevertheless, FS use is not shared with health professionals by patients despite the increased risk of herb–drug interactions, and there are very few scientific studies evaluating the use of FSs in older people or its relevance.

One path to improving patient care is improving communication between patients and health professionals. For example, patients sometimes do not know why they have to use certain drugs or experience side effects, such as loss of taste and deviating from their prescriptions, whether consciously or not, or eating less. Furthermore, the cooperation and communication between the various health professionals are not always optimal. There may be no agreements between physicians and pharmacists about the performance of drug checks. And both must reach a mutual agreement on drug checks. Any disagreement on this matter can pose a significant threat to patient safety, and optimising the exchange of information between the health professionals involved about which drugs have been prescribed, changed, or stopped is recommended as an intervention [45].

A relevant study conducted in 2006 aimed to describe the users of herbal medicine products and FSs in a community in the southeast of Sweden (aged 60+). This study concluded that physicians must consider the extensive use of herbal medicine products and FSs among elderly patients when making treatment decisions. This is because their use is reported independently of conventional medicine, since more than half of users do not discuss their use with their physicians [70].

Older adults who routinely use non-prescription therapies, including FSs or complementary and alternative medication, are at increased risk of severe drug interactions, a risk that increases with ageing and the use of specific concomitant therapies, including warfarin and aspirin. The findings of this study emphasise the importance of healthcare providers actively assessing the use of FSs/complementary and alternative medication in healthy older adults [56].

A prospective study was conducted in ambulatory patients with cancer treated with oral and/or intravenous drugs in Costa Rica. In this study, 50.3% of patients treated with anticancer agents were at risk of herbal drug interactions due to herbal supplements (FSs with herbs or herbal extracts). This study showed that a programme of medication surveillance in patients with cancer led by clinical pharmacists could prevent a relatively high proportion of patients from experiencing the potentially adverse clinical consequences of herbal drug interactions [71].

In 2021, a study was published to bring forth an up-to-date overview of essential knowledge involving the interactions between FSs and medicines relevant to health professionals’ awareness. Drugs used to treat cardiovascular, autoimmune, nervous, and oncological diseases commonly involve significant clinical interactions with FSs, several with a narrow therapeutic margin [72].

## 4. SWOT Analysis

Considering the potential toxicity of FSs to enable evidence-based advice, health professionals needs to increase their knowledge of their user populations [70].

To summarise and reflect on this more pragmatic topic, we carried out a SWOT analysis on “ Food supplements and their use in elderly subjects—challenges and risks in selected health issues “, shown in Table 2.

## 5. Conclusions and Future Directions

Concern about the healthy ageing of the population is increasingly present not only in civil society but also in political institutions worldwide. It is known that malnutrition is more prevalent among elderly polypharmacy, and this review confirms the high prevalence of polypharmacy in the elderly and that idea that the concomitant use of FSs strongly impacts several health-related outcomes. It is not the purpose of this study to assess whether or not too many drugs are prescribed to the elderly. It is believed that most of the medications administered to this age group are inevitable, so it seems that efforts should be used to reinforce the rational use of FSs. Concerns about healthy ageing lead elderly people to use other health products, particularly FSs, on their initiative. This also can explain the higher use by females. Females live longer and have, in general, more concerns about healthy ageing. Taking medicines concomitantly with FSs can trigger significant adverse reactions. Drug–FS interactions are also a relevant topic and are rarely taken into account when evaluating treatment plans. On the other hand, the use of FSs is important for treating nutritional deficiencies and improving the nutritional status of the elderly, allowing healthier ageing. Scientific evidence shows that a healthy diet helps prevent some diseases and brings economic and health benefits. Therefore, health professionals have a fundamental role in identifying the use of FSs. Another significant contribution of the current work is improving health professionals’ awareness of this issue and communication with patients. To reduce the risk of the inappropriate use of medications and FSs and prevent adverse reactions in elderly patients, it is important to implement educational interventions not only for health professionals, but also for users of healthcare products, and regulatory measures. This approach is aligned with the Global Patient Safety Action Plan 2021–2030, which aims to minimise preventable patient harm and ensure patient safety. The use of FSs is complex, and it is crucial to develop guidelines and other measures to improve the use of FSs in the elderly and assess these risks in a faster and more practical way. All these data reinforce the crucial importance of improving the regulation of FSs by experts in regulatory affairs, by industry pharmacists in the development and production of safer FSs, and by community pharmacists in advising and dispensing them.

## Figures and Tables

**Table 1:** Prevalence of polypharmacy [31,32,33,34,35,36,37].

Prevalence of Polypharmacy	Classification of Polypharmacy	Population	Country	References
25.5%	Five or more medications	People aged from 65 to 81 years with cardiovascular disease in the population of Lausanne n = 4938	Switzerland	CoLaus study, 2017 [31]
70.2%	Five or more medications daily	Patients admitted to geriatric and internal medicine acute care wards of 12 Italian hospitals	Italy	GLISTEN, 2019 [32]
		(mean age 81 years) n = 655		
44%	More than 5 drugs	Older adults between 2010 and 2013 (aged 65+ years at baseline) n = 1,742,336	Sweden	2018 [33]
28.6%	4–9 medications	Adult electronic primary healthcare records from Scotland, adults aged 60–69 years n = 180,815	Scotland	2014 [34]
6–36%	Ten or more medications	Older adults (65+ years) n = n.a. *	European Union	SIMPATHY project, 2017 [35]
30.3%	6–9 drugs	2057 older patients in emergency departments (65+ years) n = 2057	Italy	2013 [36]
56%	≥5 prescriptions within six months	Elderly 80+ years n = national cohort of 38 million Polish citizens	Poland	Kardas et al. 2018 [37]

* n.a.—not applicable.

**Table 2:** SWOT analysis on “Food supplements and elderly people—challenges and risks”.

Frames of Swot Analysis	Points of Reflection
Strengths	-Awareness and information of misguided FS use. -Eliminate adverse health effects/risks with FSs. -Contribute to the success of pharmacological therapies. -Promote the safety of polymedicated older patients and healthy ageing. -Educate or inform patients and families of patient safety incidents that cause (or could have caused) inadvertent harm. -Strengthen high-quality integrated healthcare. -Contribute to cost-effective treatment of older persons.
Weakness	-Gaps in the FS regulatory framework. -Lack of rigour in the distinction between FSs and oral nutritional supplements. -Inadequate safety evaluation of FSs. -Lack of education of physicians about food supplements -Lack of communication between patients and health professionals. -Lack of data on FS consumption in the European Union. -High complexity in establishing FS consumption patterns in older people.
Opportunities	-Inclusion in the review of the guidelines on malnutrition and the use of FSs. -Improvement of safe healthcare. -Eliminate avoidable harm in healthcare. -Improve teamwork and communication to protect patients from harm. -Assure elderly health and economic benefits. -Mitigate adverse health effects due to FSs. -Create a digital database of food supplements to support health professionals advice. -Strengthen collaboration between academia, health professionals, and the industry.
Threats	-Lack of robust FS legislation. -Lack of a harmonised definition of polypharmacy. -Unclear if polypharmacy includes or does not include FSs or oral nutritional supplements. -Difficulty in comparing prevalence data. -Hinders the definition of guidelines and health policies. -Lack of safety and quality of FSs.

## Data Availability

Not applicable.
